# Articles on Hospital Construction

**Published:** 1919-02-08

**Authors:** 


### THE NEW ERA.

### II.—Articles on Hospital Construction.

All up and down the length and breadth of our land there have sprung up, during these years of war, buildings which ministered to war's terrible needs. In fact, nothing is more creditable to the spirit of England than the speed and efficiency with which temporary hospital accommodation was supplied in every part of the country. The buildings are of many kinds. Some are mansions converted by their owners from domestic to hospital use, others are public buildings similarly converted. Others, again, are complete hospitals built, it is true, in a more or less temporary manner, but so designed as to be definitely and adequately devoted to their special purpose. The greater number consists of lightly built ward blocks attached to general or cottage hospitals, and worked from the hospital centre.

It seems to us that all these adapted buildings, of whatever class, should receive thoughtful consideration before they return to their original use. The consideration, perhaps, would not take long in the case of those princely homes in the West End of London whose owners have so generously put their possessions to public use. The palaces of Park Lane and the great houses of Belgravia, which have brought comfort and healing to many a sick and wounded soldier, must obviously return as soon as may be to their peace-time purposes.

Admirably as they have served their country in an hour of sore need, most of these buildings are not suited without further alteration for permanent hospital use.

### Houses as Permanent Places of Healing.

Other houses, notably some we can think of in the wealthier suburbs, have been so transformed, often at great expense, by their liberal-minded owners, that one almost wonders how they will ever settle down again to the functions of an ordinary home. Some of these, if their possessors were so minded, might, one thinks, with very little adaptation (or none) become permanent places of healing by being taken over as convalescent homes or small isolation hospitals. One cannot suggest this as a general policy; but the matter is at least worth a thought.

The buildings with which this article is mainly concerned are the temporary wards erected in the grounds of general hospitals. What is to become of them? Are they of further use?

It must be at once admitted that in some cases of which we have knowledge these temporary expedients have been crowded on to the ground with a density of surface occupation which nothing but the acute stress of war could be held to justify. We have a case in mind where a comparatively small breathing space at the back of an already rather stuffy London building has been crammed to the utmost limit with additional wards. In this case the buildings must obviously be scrapped. But when? No hospital, we venture to think, should get rid of its temporary wards without at least considering whether they cannot be made serviceable during some needful building or renewal in the main fabric. The great difficulty in conducting building operations or even thorough cleaning and redecoration in a hospital is the problem of the disposal of, or suppression of, the patients. The superfluous accommodation now available offers a golden opportunity for such work, which should on no account be allowed to slip by without thought.

### After-use for Military Wards.

Other hospitals have built their temporary wards in a rather less temporary manner. One small country hospital which was in sore need of additional sleeping accommodation for nurses hit upon the idea of instructing its architect to design its military ward in such a way that it could be readily converted on disuse into a suite of nurses' rooms complete with bath and sanitary accommodation. Every detail, position of windows, situation of radiators, &c., was thought out with this intention and the building is now ready for conversion. Even in cases where no such provision has been made, it is likely that the soldier-wards might possibly be converted into some much-needed requirement of the hospital. This, of course, could not be the case where the method of construction is merely of the short-lived type. One point must be borne in mind whenever it is proposed to use such wards as *wards*. In most instances the military accommodation has been set out on a basis of space which allows much less than the normal and healthful superficial and cubic space per bed. Such spacing, essential under war pressure, but deleterious in days of peace, should on no account be perpetuated.

In all cases it would be desirable to take architectural advice on the adequacy of the buildings for the strain of prolonged existence. Walls of thin asbestos sheeting are not necessarily suited for permanent occupation; nor would the still prevailing by-laws, which it is to be hoped will be amended throughout England, permit their retention as external walls. Roofs and roof supports have also in many places assumed exiguous dimensions, and flimsy materials which are not calculated to stand the wear and weather of prolonged existence have been used.

But with all such reservations, it is still well worth while for hospital owners to consider before they send their military wards to limbo whether the interests of their institution may not be usefully served by a retention or remodelling or a temporary continuance of these swiftly constructed shelters which have played so noble a part in the relief of the sufferings of war.

☞ The previous article appeared on January 25, p. 354.

